# Survival Analysis and Risk for Progression of Intraductal Papillary Mucinous Neoplasia of the Pancreas (IPMN) Under Surveillance: A Single-Institution Experience

**DOI:** 10.1245/s10434-016-5661-x

**Published:** 2016-11-07

**Authors:** Marco Del Chiaro, Zeeshan Ateeb, Marcus Reuterwall Hansson, Elena Rangelova, Ralf Segersvärd, Nikolaos Kartalis, Christoph Ansorge, Matthias J. Löhr, Urban Arnelo, Caroline Verbeke

**Affiliations:** 1 Pancreatic Surgery Unit, Division of Surgery, Department of Clinical Science, Intervention and Technology (CLINTEC), Karolinska Institutet at Center for Digestive Diseases Karolinska University Hospital, Stockholm, Sweden; 2 Division of Radiology, Department of Clinical Science, Intervention and Technology (CLINTEC), Karolinska Institutet at Karolinska University Hospital, Stockholm, Sweden; 3 Department of Pathology, Karolinska University Hospital, Stockholm, Sweden

## Abstract

**Purpose:**

While surveillance of the majority of patients with IPMN is considered best practice, consensus regarding the duration of follow-up is lacking. This study assessed the survival rate and risk for progression of IPMN under surveillance.

**Methods:**

All patients diagnosed with and surveyed for IPMN between January 2008 and December 2013 were identified and assigned to two groups: patients without indication for surgery (Group 1), and patients whose IPMN required surgery but were inoperable for general reasons (Group 2). Disease progression and survival data were compared between both groups.

**Results:**

In total 503 patients were identified, of whom 444 (88.3%) were followed up. Group 1 included 395 patients, and Group 2 had 49. In Group 1, IPMN-specific 1-, 5-, and 10-year survival rates were 100, 100, and 94.2%, respectively. Four patients died of associated or concomitant pancreatic cancer, and 230 patients (58.2%) experienced disease progression. The 1-, 4-, 10-year cumulative risk for progression and for surgery was 11.2, 70.6, 97.5, and 2.9, 26.2, 72.1%, respectively. In Group 2, the 1-, 5-, 10-year IPMN-specific survival rate was 90.7, 74.8, and 74.8%, respectively.

**Conclusions:**

This study confirmed the safety of surveillance for patients with IPMN who do not require surgery. However, the risk for disease progression and for surgery increases significantly over time. The study results support International and European guidelines not to discontinue IPMN surveillance and validate the European recommendation to intensify follow-up after 5 years. The fairly good prognosis of patients whose IPMN requires surgery but cannot undergo resection suggests a relatively indolent disease biology.

Intraductal papillary mucinous neoplasm (IPMN) of the pancreas is a disease with a high prevalence. It is estimated that the prevalence of pancreatic cystic neoplasms (PCNs) in the general population is approximately 20–30% and that half of these are IPMNs.^1^,^2^ While it is recognized that IPMNs can progress to cancer, the high prevalence of IPMNs and the low incidence of pancreatic cancer (PC) in the general population indicate that only a minority of IPMNs progress to invasive carcinoma.^3^ Considering that IPMN of the pancreas was defined by the World Health Organization only as recently as in 1996, there is a lack of information regarding the natural history of these neoplasms and in particular regarding long-term prognosis.^4^ Published data show that the risk for cancer differs according to the epithelial type of IPMN and that it is higher in IPMNs affecting the main pancreatic duct, either in isolation or combined with branch duct involvement (main-duct or mixed-type IPMNs, respectively). For this reason, current guidelines recommend surgical resection for all IPMNs with involvement of the main pancreatic duct, provided the patient is fit for surgery.^5^,^6^ In contrast, the risk for cancer is significantly lower for IPMNs that are limited to branch ducts (branch-duct IPMN); therefore, surgical resection is considered to be an indication in only a minority of patients with branch-duct IPMN. However, because it is currently not possible to predict which of the IPMNs that do not require surgical resection will progress to cancer, a large proportion of patients require life-long follow-up.^5^,^6^ Data from the literature support the current conservative management of the majority of patients with “small” (<3–4 cm) branch-duct IPMNs, who do not have IPMN-related symptoms and do not present worrisome radiological features. Invasive carcinoma is reported to develop during follow-up in 1–11% of patients.^7^–^9^ The risk for pancreatic cancer developing at a distance from the IPMN also seems to be increased, with a 5-year incidence reported in approximately 7% of patients under surveillance and a standardized incidence ratio of 15.8- to 26-fold.^10^,^11^ Even though published data support the safety of surveillance for the majority of patients with IPMN, experiences are not uniformly positive.^12^ This is most likely explained by the use of different inclusion criteria and different indications for surgery or surveillance as well as by the small size of some of the study cohorts.

 Currently, IPMNs represent the most promising opportunity for the prevention of pancreatic cancer through the establishment of surveillance programs and the treatment of precancerous lesions. However, surveillance of patients with IPMN incurs high healthcare costs and bears the risk for overtreatment or undertreatment of patients affected by the disease.^13^,^14^ For this reason, analysis of large series is needed to clarify the value and safety of patient surveillance.

The main purpose of this study was to analyze the survival rate and risk for progression in a large series of patients who are under surveillance for IPMN that according to current guidelines does not require surgical resection. The secondary purpose of the study was to analyze the safety of conservative management and the need for long-term follow-up of this patient group.

## Methods

This study was based on a prospectively collected cohort of patients who were diagnosed with IPMN at the Pancreas Unit at Karolinska University Hospital between January 2008 and December 2013. Also included were patients who were surveyed during the study period but whose diagnosis of IPMN had been established before January 2008. An absolute inclusion criterion for the study cohort was a diagnosis of IPMN based on cytological confirmation and/or radiological demonstration of a cystic lesion that communicated with the main pancreatic duct.

Data were retrospectively analyzed after approval from the local Ethical Committee was obtained. All patients were managed according to the Sendai criteria^15^ (until October 2011) and the European guidelines criteria^5^ (from November 2011). Excluded from the series were patients with an IPMN that fulfilled the criteria for resection and underwent surgery directly after discussion at the multidisciplinary conference. Further excluded were patients who were diagnosed with locally advanced or metastatic IPMN-associated cancer. The remaining patients were assigned to two groups. Patients who were enrolled in the surveillance program in accordance with the Sendai criteria (until October 2011) or the European guidelines (from November 2011) formed Group 1. Group 2 included patients who were not eligible for surgery for general reasons and therefore remained under follow-up, although their IPMN fulfilled indications for surgery.

### Indications for Surgery

Until November 2011, we used the indications for surgery in patients with IPMN as suggested by the Sendai criteria.^15^ When the European guidelines were published in November 2011, the recommendations of these guidelines were adopted by our hospital.^5^ In patients with BD-IPMN, surgery was indicated in case of symptomatic disease (jaundice, acute pancreatitis related to IPMNs) and/or in case one or multiple of the following features were detected: maximum cyst diameter ≥4 cm, mural nodules, rapid increase in cyst size, elevated serum levels of Ca 19-9. Patients with a main pancreatic duct >6 mm were considered being affected by MD-IPMN and represented a surgical indication.^5^

### Diagnostic Workup and Follow-up

All patients included in the study were discussed at the pancreatic multidisciplinary conference at Karolinska University hospital (including dedicated radiologists, surgeons, gastroenterologists, pathologists, endoscopists, and oncologists). The diagnosis of IPMN was reached by using a single conventional diagnostic modality (CT scan, MRI) or a combination of these. EUS + FNA was used in case of nondiagnostic, conventional radiology (2nd level examination). In selected cases, a combination of multiple investigational modalities was used to establish the diagnosis. Follow-up of the patient cohort consisted of MRI every 6 months during the first year, once per year in the following 4 years, and thereafter again every 6 months, as recommended by the European guidelines.^5^ Other imaging modalities were used for patients with contraindications for MRI.

### Statistical Analysis

Comparison of continuous variables was performed by a *t* test. Comparison of categorical variables was done by *χ* ^2^ analysis. Risk assessment and survival were evaluated with Kaplan–Meyer analysis using Graph Pad Prism software©.

## Results

Between January 2008 and December 2013, 503 patients with a radiological or histological diagnosis of IPMN were discussed at the pancreatic multidisciplinary conference at Karolinska University Hospital. Of these, 51 patients (10.1%) went straight to surgery for resection of an IPMN that fulfilled the criteria for surgical treatment. Another 49 patients (9.7%) with a “surgical IPMN” did not undergo surgery because of poor fitness. Eight patients (1.6%) were diagnosed with locally advanced or metastatic IPMN-associated cancer at the time of presentation and were referred for chemotherapy. The remaining 395 patients with a “non-surgical IPMN” were enrolled in a surveillance program. The current study is based on the latter patient group (Group 1) and the group of 49 patients with contraindications for surgery, who were also kept under surveillance (Group 2).

### Group 1: Patients Under Surveillance in Accordance with the Guidelines

**Fig. 1:**
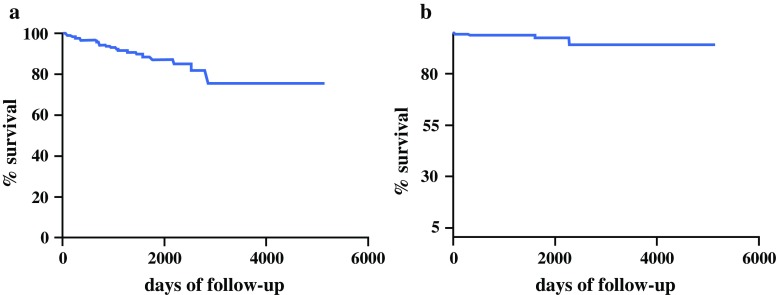
**a** Actuarial survival of patients with IPMN in Group 1. **b** IPMN-specific actuarial survival of patients in Group 1

**Fig. 2:**
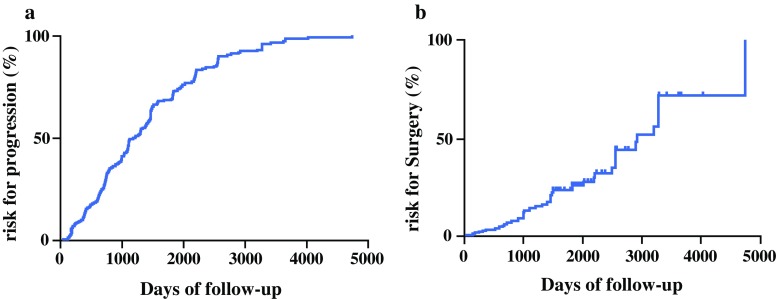
**a** Cumulative risk for progression of IPMN during the surveillance period. **b** Cumulative risk for surgery for IPMN progression during the surveillance period

Of the 395 patients included in this group, 194 (49.1%) were males and 201 (50.9%) females. Median age at the time of discussion at the multidisciplinary conference was 67.3 (range 18–93) years. Median follow-up time was 932 days (range 180–5110 days). All patients included in this group were diagnosed with branch-duct IPMN, and 49 of them (12.4%) also had a prominent main pancreatic duct (defined as a diameter ≥4 mm and <6 mm). Radiological localization of the IPMN was multifocal in 138 cases (34.9%), in the uncinate process in 126 (31.9%), pancreatic tail in 53 (13.4%), pancreatic head in 41 (10.4%), and pancreatic body in 37 (9.4%). Twenty-five patients (6.3%) had a family history of pancreatic cancer. The median diameter of the cysts was 20 mm (range 8–40 mm). Thirty-three patients (8.3%) died during surveillance, of whom four (1%) due to progression of the IPMN to cancer (2 patients) or the development of concomitant pancreatic cancer, i.e., cancer separate from the IPMN (2 patients). The cause of mortality during follow-up was IPMN with associated or concomitant cancer (each in 2 patients, 0.5%), extrapancreatic cancer (*n* = 5, 1.3%), and other noncancer-related disease (*n* = 24, 6%). The overall 1-, 5- and 10-year survival rates of patients in Group 1 were 100, 99.7, and 75.4%, respectively (Fig. 1a). The IPMN-specific 1-, 5-, and 10-year survival rates were 100, 100, and 94.2%, respectively (Fig. 1b). A total of 230 patients (58.2%) experienced disease progression during follow-up. The risk for progression at 1, 5, and 10 years after the initial diagnosis of IPMN was 11.2, 70.6, and 97.5%, respectively (Fig. 2a; Table 1).

**Table 1:** Incidence and risk for progression and for surgery in patients under follow-up for IPMN (Group 1, *n* = 395)

Follow-up (years)	No. of patients remaining under FU	Cumulative risk for progression (%)	Cumulative risk for surgery (%)
1	269	11.2	2.9
2	184	29.0	6.7
3	123	45.6	13.8
4	84	61.7	21.4
5	58	70.6	26.2
6	33	81.6	30.0
7	21	88.8	44.3
8	13	92.5	52.2
9	11	95.6	72.1
10	5	97.5	72.1

*FU* follow-up

Progression of an IPMN was characterized by an increase in (>1 mm) size (186 patients; 47.1%), dilatation of the main pancreatic duct (27 patients; 6.8%), dilatation of the main pancreatic duct in addition to worrisome radiological features (1) (6 patients; 1.5%), radiological worrisome features only (6 patients; 1.5%), increase in cyst size in addition to radiological worrisome features (2 patients; 0.5%), and IPMN-related symptoms, e.g., acute pancreatitis or jaundice (3 patients; 0.8%). The median increase in cyst size during the period of follow-up was 3.7 (range 2–30) mm. Mean age at the time of diagnosis, gender, a positive family history of pancreatic cancer, multifocality, or a cyst diameter >30 mm were not associated with an increased risk for progression. In contrast, both prominence of the main pancreatic duct (diameter ≥6 mm) and localization in the head of the pancreas (incl. uncinate process) were associated with a significantly increased risk for progression (Table 2).

**Table 2:** Comparison of patient- and IPMN-related characteristics between patients with and without IPMN progression (Group 1, *n* = 395)

Variables	No. of patients with progression (%) (*n* = 230)	No. of patients without progression (%) (*n* = 165)	*P*
Mean age (years)	65.9 ± 0.8	67.7 ± 0.9	0.1
Male gender	103 (44.8)	91 (55.2)	0.05
Positive family history of PC	17 (7.4)	8 (4.8)	0.4
MPD diameter ≥6 mm	46 (20.0)	3 (1.8)	<0.0001
Multifocal IPMN	72 (31.3)	66 (40.0)	0.08
Cyst diameter ≥30 mm	44 (19.1)	37 (22.4)	0.4
Localization: head of pancreas (incl. uncinate process)	109 (47.4)	58 (35.1)	0.01

*PC* pancreatic cancer, *MPD* main pancreatic duct

Overall, in 55 patients (13.9%) of Group 1, progression of the disease was clinically relevant, because the IPMN required surgical resection. The indications for surgery in these patients were: increase in cyst dimension only (*n* = 11, 20.0%) and combined with worrisome features (*n* = 2, 3.6%), dilatation of the main pancreatic duct (≥6 mm) in isolation (*n* = 27, 49.2%) and combined with radiologic worrisome features (*n* = 6, 10.9%), radiologic worrisome features (6, 10.9%), and IPMN-related symptoms (new onset of jaundice or acute pancreatitis without any other cause; *n* = 3, 5.4%).

The median time from diagnosis to surgery was 48.2 (range 6–158) months. The cumulative risk for surgery at 1, 5, and 10 years was 2.9, 26.2, and 72.1%, respectively (Table 1; Fig. 2b).

Histology of the surgical specimen confirmed the necessity of surgery in 47 of the 55 patients (85.4%) who underwent surgical resection. Twenty-nine patients (52.7%) had a mixed-type IPMN with intermediate- or high-grade dysplasia, 10 (18.2%) had a branch-duct IPMN with high-grade dysplasia, 4 (7.3%) a main-duct IPMN with intermediate- or high-grade dysplasia, 2 (3.6%) ductal carcinomas located separate from the IPMN, 2 IPMN (3.6%) with transition into invasive adenocarcinoma, whereas 8 patients (14.5%) had a branch-duct IPMN with low-grade dysplasia.

### Group 2: Patients Under Surveillance Due to General Contraindications for Surgery

**Fig. 3:**
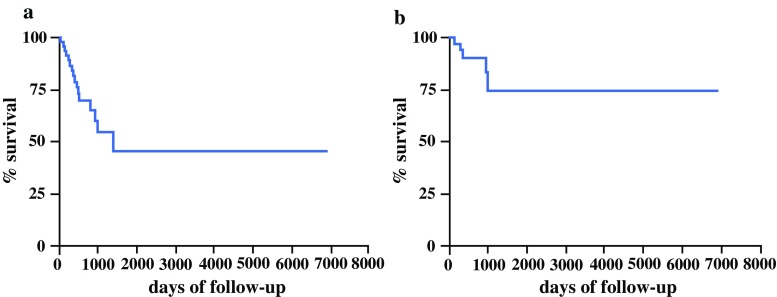
**a** Actuarial survival of patients with IPMN in Group 2. **b** IPMN-specific actuarial survival of patients in Group 2

Forty-nine patients with an IPMN that fulfilled the criteria for surgical resection according to the guidelines, but who were not fit for surgery or objected to surgery, were followed-up during the study period.^1^,^2^ Median patient age was 75.1 (range 50–93) years. Twenty-seven (55.1%) were males and 22 (44.9%) females. The median follow-up time was 775 (range 90–2190) days. The tumor features that represented a surgical indication were main-duct IPMN in 13 patients (26.5%), mixed-type IPMN in 27 (55.1%), branch-duct IPMN with a diameter >40 mm in 6 (12.3%), and branch-duct IPMN with worrisome radiological features in 3 (6.1%). The 1-, 5-, and 10-year overall survival rate for these patients was 74.8, 40.6, and 40.6%, respectively (Fig. 3a). The IPMN-specific 1-, 5-, and 10-year survival rates were 90.7, 74.8, and 74.8%, respectively (Fig. 3b).

## Discussion

This study represents one of the largest, single-institution analysis of the results of a surveillance program for patients with IPMN, who were managed according to current guidelines.^5^,^6^ Our study was one of the few to report on the outcome of patients whose IPMN required resection but who could not undergo surgery due to general contraindications. The study results support the recommendations of current guidelines by demonstrating that the majority of patients with branch-duct IPMN (78.5% in this study) can be managed safely with a conservative approach.^5^,^6^ The mortality rate due to the development of IPMN-associated cancer or concomitant pancreatic cancer separate from the IPMN is 1% in the cohort of patients who were under surveillance as recommended by the guidelines. This does not exceed the mortality rate associated with pancreatic surgery, even in the most experienced centers. Interestingly, the cumulative risk for IPMN progression increased over time and was 70% at 5 years and 97.5% at 10 years from the time of detection of the IPMN. Even the cumulative risk for progression to an IPMN that required surgery increased continuously and reached 26% at 5 years and 72.1% at 10 years of follow-up. Interestingly, the percentage of patients with an IPMN that developed features requiring surgery increased significantly also after 5 years of follow-up. The median time from first diagnosis of an IPMN (not requiring resection) to surgery was 48 months. The final histology of IPMNs that progressed and required surgery confirmed that the large majority of these lesions (85.4%) represented a true surgical indication, either with respect to the IPMN type (main-duct or mixed-type) or the grade of dysplasia (high-grade dysplasia to invasive carcinoma). This indicates that the detection of changes in cyst characteristics or in patient symptoms is indeed more predictive than a simple “one shot” evaluation at the time of first diagnosis. The observations in this study demonstrate that progression of IPMNs that do not fulfill criteria for surgery to lesions that require resection can occur even after 5 years or more following diagnosis. These observations validate and support the recommendations of the European and International guidelines regarding the necessity of a life-long follow-up of these patients. Moreover, the findings of the current study do not support the recent statement from the American Gastroenterological Association (ASA), which suggests that surveillance does not need to be continued for cysts without significant change after 5 years of follow-up.^5^,^6^,^16^ Considering that both the risk for progression and the risk for surgery gradually increase over time, our results support also the recommended strategy of a low frequency of control investigations in the initial years of the screening program, followed by an intensified protocol after 5 years.^5^ The results of this study further show that radiological prominence of the main pancreatic duct (diameter ≥6 mm) is associated with an increased risk for progression. This observation confirms the importance and prognostic significance of even mild pancreatic duct dilatation, as recently reported by Hackert et al. and stated by the European guidelines.^5^,^17^,^18^ Furthermore, the current analysis shows that localization of an IPMN in the pancreatic head also is associated with an increased risk for progression. This finding seems to confirm previous observations by Ammori et al.^19^.

A limitation of our study is the relatively small number of patients with a long (more than 5 years) follow-up. As a consequence, the study results do not represent strong evidence on which recommendations for follow-up after 10 years could be based. Nonetheless, the results of this study support long-term follow-up rather than surveillance limited to 5 years. Although comparison of existing guidelines is beyond the scope of this study, it is important to note that a significant number of patients whose IPMN progressed during the study period to the point of requiring surgery would not have been identified if follow-up had been performed according to the ASA guidelines. The related problems of societal costs associated with this strategy require further investigation, especially considering the high prevalence of IPMN in the general population. New biomarkers and/or less expensive surveillance modalities will probably partially overcome this problem in the near future.^20^ Finally, our study confirmed previous reports that patients with an IPMN that formally requires surgery but who cannot be operated because of general contraindications, have a relatively high IPMN-specific survival.^21^,^22^ This observation opens a new field of potential investigation, exploring for example whether these patients would benefit from treatment with ablative techniques or similar modalities for symptomatic palliation.^23^,^24^
